# High power laser in chordae tendineae to improve heart mitral regurgitation: an experimental study in swine

**DOI:** 10.1186/1749-8090-8-S1-O282

**Published:** 2013-09-11

**Authors:** NC Pinto, PMA Pomerantzeff, A Deana, D Zezell, RL Marcos, RABL Martins, VD Aiello, FB Jatene, LA Lopes, MC Chavantes

**Affiliations:** 1 Cardiovascular Surgery Department, Heart Institute (InCor), Hospital das Clínicas da Faculdade de Medicina da Universidade de São Paulo, São Paulo, Brazil; 2 Universidade de São Paulo, São Paulo, Brazil; 3 Nuclear and Energetic Research Institute, University of São Paulo, São Paulo, Brazil; 4 Post Graduate Program Biophotonics Applied to Health Sciences, Nove de Julho University, São Paulo, Brazil; 5 Pathology Department, Heart Institute (InCor), Hospital das Clínicas da Faculdade de Medicina da Universidade de São Paulo, São Paulo, Brazil; 6 Research and Education Center for Photo Therapy in Health Sciences, São Carlos, Brazil

## Background

Rheumatic fever remains a significant worldwide cause of mitral regurgitation, responsible for approximately 90% of early childhood valvular surgery in Brazil. Elongated/flail chordate are frequently responsible for such condition that must be surgically corrected.Despite recent surgical progress in mitral valve reconstruction techniques, there are no published reports shortening the chordate tendineae applying Surgical Laser.The purpose of this study was to analyze and compare the histological tissue mitral valve chordae and its mechanical resistance with and without High Power Laser (HPL) application.

## Methods

A total of 20 porcine mitral valve chordae were measured and divided in 2 groups: Control Group (GI): chordae without HPL and Laser Group (GII): chordae submitted to HPL procedure.Diode CW Laser application under controlled conditions with following parameters: λ=980nm, P=3W, T= 15-25s, E= 30-60J was performed.The chordae temperature was controlled in real time by ultra sensible thermography equipment. A testing machine was used to measure the chordae tensile properties and histological analysis was carried out.

## Results

Histological analysis showed in GI the presence of usual collagen bundles organized arrangement, while in GII, after the temperature range (43oC to 46oC) has been reached, the collagen bundles were organized differently with significant chordae tendineae reduction.Our findings show a change of chordae tendineae’s resistance in laser group - GII (greater elasticity).

## Conclusion

Based on our experimental preliminary data,it suggests that the application of right laser parameters may assist to treat valve insufficiency.Although more studies are needed to verify this method usefulness.The laser application provides a promising outcomes and it appears to have a unlimited potential for cardiac surgery, especially in human pathological valves with cost-effectiveness.

